# Molecular Insights into Therapeutic Potentials of Hybrid Compounds Targeting Alzheimer’s Disease

**DOI:** 10.1007/s12035-022-02779-6

**Published:** 2022-03-26

**Authors:** Ankit Jana, Arkadyuti Bhattacharjee, Sabya Sachi Das, Avani Srivastava, Akshpita Choudhury, Rahul Bhattacharjee, Swagata De, Asma Perveen, Danish Iqbal, Piyush Kumar Gupta, Saurabh Kumar Jha, Shreesh Ojha, Sandeep Kumar Singh, Janne Ruokolainen, Niraj Kumar Jha, Kavindra Kumar Kesari, Ghulam Md Ashraf

**Affiliations:** 1 School of Biotechnology, Kalinga Institute of Industrial Technology (KIIT) Deemed To Be University, Campus-11, Patia, Bhubaneswar, Odisha 751024 India; 2 Department of Pharmaceutical Sciences and Technology, Birla Institute of Technology, Mesra, Ranchi, Jharkhand 835215 India; 3 Department of English, DDE Unit, The University of Burdwan, GolapbagBurdwan, West Bengal 713104 India; 4 Glocal School of Life Sciences, Glocal University, Mirzapur Pole, Saharanpur, Uttar Pradesh India; 5 Department of Medical Laboratory Sciences, College of Applied Medical Sciences, Majmaah University, Al-Majmaah, 11952 Saudi Arabia; 6 Department of Life Sciences, School of Basic Sciences and Research (SBSR), Sharda University, Greater Noida, Uttar Pradesh 201310 India; 7 Department of Biotechnology, School of Engineering and Technology (SET), Sharda University, Greater Noida, Uttar Pradesh 201310 India; 8 Department of Pharmacology and Therapeutics, College of Medicine and Health Sciences, United Arab Emirates University, 15551 Al Ain, United Arab Emirates; 9 Department of Applied Physics, School of Science, Aalto University, 00076 Espoo, Finland; 10 Pre-Clinical Research Unit, King Fahd Medical Research Center, King Abdulaziz University, Jeddah, Saudi Arabia; 11 Department of Medical Laboratory Technology, Faculty of Applied Medical Sciences, King Abdulaziz University, Jeddah, Saudi Arabia

**Keywords:** Alzheimer’s disease, Pathogenesis, Neuronal molecular targets, Cellular pathways, Targeted hybrid therapeutics

## Abstract

Alzheimer’s disease (AD) is one of the most complex progressive neurological disorders involving degeneration of neuronal connections in brain cells leading to cell death. AD is predominantly detected among elder people (> 65 years), mostly diagnosed with the symptoms of memory loss and cognitive dysfunctions. The multifarious pathogenesis of AD comprises the accumulation of pathogenic proteins, decreased neurotransmission, oxidative stress, and neuroinflammation. The conventional therapeutic approaches are limited to symptomatic benefits and are ineffective against disease progression. In recent years, researchers have shown immense interest in the designing and fabrication of various novel therapeutics comprised of naturally isolated hybrid molecules. Hybrid therapeutic compounds are developed from the combination of pharmacophores isolated from bioactive moieties which specifically target and block various AD-associated pathogenic pathways. The method of designing hybrid molecules has numerous advantages over conventional multitarget drug development methods. In comparison to in silico high throughput screening, hybrid molecules generate quicker results and are also less expensive than fragment-based drug development. Designing hybrid-multitargeted therapeutic compounds is thus a prospective approach in developing an effective treatment for AD. Nevertheless, several issues must be addressed, and additional researches should be conducted to develop hybrid therapeutic compounds for clinical usage while keeping other off-target adverse effects in mind. In this review, we have summarized the recent progress on synthesis of hybrid compounds, their molecular mechanism, and therapeutic potential in AD. Using synoptic tables, figures, and schemes, the review presents therapeutic promise and potential for the development of many disease-modifying hybrids into next-generation medicines for AD.

## Introduction

Alzheimer’s disease (AD) is one of the progressive neurodegenerative disorder characterized by brain cognitive impairment which influence speech, behavior and motor function and is most common form of dementia [1]. Since the recognition of the first case of AD by Alois Alzheimer (a German psychiatrist) in 1906, the significant progress has been made in understanding the cellular and molecular mechanisms on pathogenesis of AD [2, 3]. It is prevalent among elderly people, mostly belonging to age ≥65, that comprises one of the significant parts of the population. Ageing is one of the major risk factor and there are no specific feature of diagnosis except pathological diagnosis. AD prompts a poor prognosis with a low chance of early detection [4]. It is pathologically characterized by extracellular amyloid plaque and intracellular neurofibrillary tangles (NFTs) constituting hyperphosphorylated tau-protein deposited in human brain tissues [ 5, 6]. The census data (1900–2050) have shown that the population of the people belonging to this group are growing rapidly, therefore AD is becoming a matter of concern, mostly due to high cost of conventional therapeutics and unattainability of targeted therapeutics [4]. As of now, AD is considered one of the debilitating and devastating disease in the ageing population and represent a therapeutic enigma with being completely incurable [7]. The conventional therapeutics available for the management of AD mainly includes cholinesterase inhibitors (rivastigmine, galantamine, donepezil, and tacrine) that effectively shows benefits in a set of symptoms and target late aspects of AD. Among many approaches, the most effective approach includes inhibiting or preventing the aggregation of Aβ (β-amyloid) plaques in the brain cells [8]. Because of reduced absorption in neuronal cell membranes, these conventional agents mainly affect progression of the disease [9, 10]. There is still necessity of the drugs which can offer a cure for the disease. Despite significant advances in understanding the pathogenesis of AD, still there is scarcity of knowledge regarding the exact molecular mechanism which limit development of targeted and effective therapy. The recognition of targets is essential for intervention to prevent the onset and development of disease as well as direct therapy for the reversal of the modifications in the brain tissue [11, 12]. Numerous investigations have demonstrated linkage between several factors which leads to the onset of AD and hypertension appears one of the most common contributing factor leads to increased plaque formation in the brain [13–15]. Homocysteine, a sulfur-containing amino acid, contribute in the development of Aβ plaques and plays a crucial role in the pathogenesis of AD [16, 17]. Moreover, high sugar levels and obesity also exhibited potential associations with some non-conformities to the risk of developing AD [18, 19].

The single drugs or bioactives available for the treatment of AD have exhibited insufficient effectiveness owing to the polygenic etiology of AD. Therefore, the focus of the researchers and neuroscientists has shifted to “one molecule-multi targeting” molecules, more precisely recognized as hybrid therapeutic compounds, which are known to consist two or more than two bioactive moieties with complementary pharmacophoric and pharmacological properties facilitating a synergistic effect [20–22]. These engineered molecules, often defined as “multi-target-directed ligands” (MTDLs), enable simultaneous delivery of the bioactive moieties to the targeted organs with superior targeting, reduced drug-drug interactions, and lower drug resistance, leading to negligible toxicity and low-cost preclinical trials [20, 23]. In this review, we comprehensively discussed the contributing factors, molecular pathogenesis and development of targeted hybrid therapeutic compounds for the better management of AD.

### Molecular Pathogenesis of AD

**Fig. 1:**
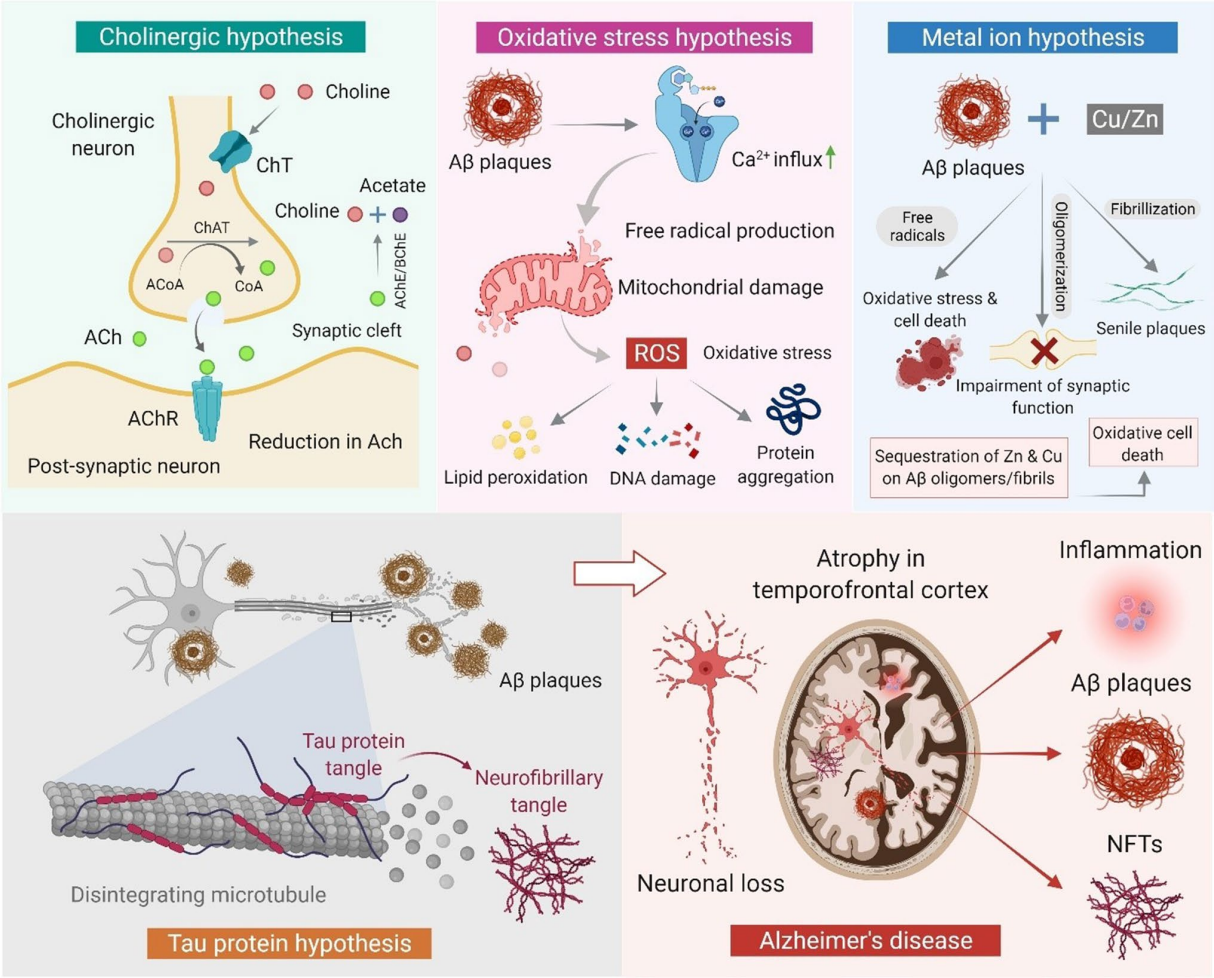
Schematic showing various hypotheses and pathomechanism of AD

The etiopathogenesis of AD is primarily characterized by the accumulation of two protein markers: extracellular Aβ plaques and NFTs of hyperphosphorylated tau protein (Fig. 1) [5, 6]. Additionaly, other hypotheses, including the loss of synapses, oxidative stress, and nerve cell death are recognized among the pathogenic players and are frequently observed to coexist [24–26].

In late 1970s, the researchers mainly focused on identifying the contribution of two putative enzymes: acetylcholinesterase (AChE) and butyrylcholinesterase (BChE) responsible for the hydrolysis of cholinergic neurotransmitters, and demonstrated their potential roles in AD therapy [27–29]. For effective AD therapy, dual inhibition of AChE and BChE has been endorsed to provide a superior therapeutic benefit, few specific behavioral benefits, and the avoidance of AChE upregulation. This investigation, thus, gradually shifted the attention of the researchers towards developing dual inhibitors (AChE and BChE) to avoid the inefficacy of AChE [1]. Another etiological characteristic of AD is the amyloid cascade, which centers on the accumulation of the Aβ peptide (Fig. 1) in the brain parenchyma. The aberrant synthesis of amyloid precursor protein (APP) by β-secretases and γ-secretases produces Aβ_40_ and Aβ_42_ monomers respectively, which oligomerize into biochemically insoluble fibrils and aggregate within the Aβ plaques [30]. These plaques block proteosome functions, alter intracellular calcium (Ca^2+^) levels, and limit the mitochondrial activities, leading to increased neurotoxicity and fibrilization rate [31]. Tau protein, another hallmark of AD (Fig. 1), is a microtubule-associated protein that accomplishes the role of stabilizing microtubules. The hyperphosphorylated tau protein produces an imbalance and deteriorates the axonal transport of the APP in nerve cell bodies by forming NFTs. Further, the collapse of microtubules causes interruptions in the neuronal communications lead to the neuronal cell death [32].

The central nervous system (CNS) is secured by the blood–brain barrier (BBB) and the cerebrospinal fluid (CSF) barrier [33]. The proteins through membrane transporters at the BBB are discarded into two major categories, named as uptake and efflux transporters. Moreover, at the level of BBB many uptake transporters play significant role in transporting solutes through circulation into the endothelial cells and then into the brain cells/tissues across the basolateral membranes. Also, the BBB regulates both inward and outward movement of biomolecules to and from the brain neuronal system and contributes to the transport of Aβ to the brain. Thus, BBB plays an important function in the pathogenesis of AD by regulating the aggregation of Aβ peptides within the brain tissue. These cellular processes makes BBB as one of the most important targets that need to be studied in order to improve the delivery and targetability of the drugs to the brain [34, 35]. The CNS is easily susceptible to oxidative stress which is considered one of the major contributors to the neuronal cell death in AD. Monoamine oxygenase (MAO) targets the enzymatic metabolism of neurotransmitters by altering the level of lipids, proteins, and DNA and suppressing respiration. The abundance of Cu^2+^ and Fe^2+^ potentiate several reactive oxygen species (ROS) and reactive nitrogen species (RNS) leading to Aβ neurotoxicity. Aβ aggregation also increases the production of ROS (Fig. 1) and causes oxidative stress in mitochondria [36, 37].

The “neuroinflammatory hypothesis” which serves as the first layer of protection in the event of an injury or infection, frequently responds disproportionately and injures brain cells further leads to rapid neurodegeneration [38]. Ca^2+^ signaling plays an important role in many intracellular and extracellular activities. Ca^2+^ triggers synaptic vesicle exocytosis, essential for synaptic transmission, thus releases the neurotransmitters [39]. Ca^2+^ also manipulates the cytoskeleton and associated proteins aiding neuroplasticity and neuronal development. However, prolonged elevation of cytosolic Ca^2+^ levels triggers the activation of several kinase-dependent signaling cascades that are significantly responsible for the generation of ROS and loss of synaptic plasticity [40]. In this event, the aggregation of MAO-B observed high around senile plaques, leads to the pathogenesis of AD. The catalytic properties of MAO produce H_2_O_2_ inside reactive microglia in brain tissue and trigger the onset of oxidative stress and consequent degeneration of the brain cell [41].

### Significant Role of Hybrid Therapeutic Compounds in AD

The complex etiology of AD has reinvigorated the development and establishment of novel therapeutic strategies comprised of multitargeted therapeutics targeting specific diseased sites in the brain. Because of the ambiguity, compounds targeting single molecular targets have exhibited limited impact over the complicated pathogenic networks. Till date, studies have shown that various pathogenic pathways are responsible for the initiation and progression of AD, and they are also associated with various cellular response and sequence of events that deteriorate the diseased state while exponentially enhances the chances neuronal death. In many instances, the “one-target one-molecule” strategy is found to be ineffective in influencing the pathogenic events, resulting in limited clinical benifits. Therefore, one single moiety comprised of specific targets focusing important molecular sites could be the most effective method for establishing a potential therapeutic strategy in AD therapeutics. As compared to co-treatment or “single pill” medication combos, these targeted strategies have exhibited substantial benefits as demonstrated by reduced adverse effects of drugs/bioactives and improved pharmacokinetic as well as pharmacodynamic profile with ADME optimization. However, the advancements of “multi-target-directed ligands” continue to be a challenge that need to be addressed in a variety of ways [42]. The most common method is to develop combinatorial therapeutic approaches by combining numerous therapeutic active moieties to form a single hybrid complex. Combining two or more therapeutic active compounds into a single hybrid complex is considered to be one of the typical strategy.

**Fig. 2:**
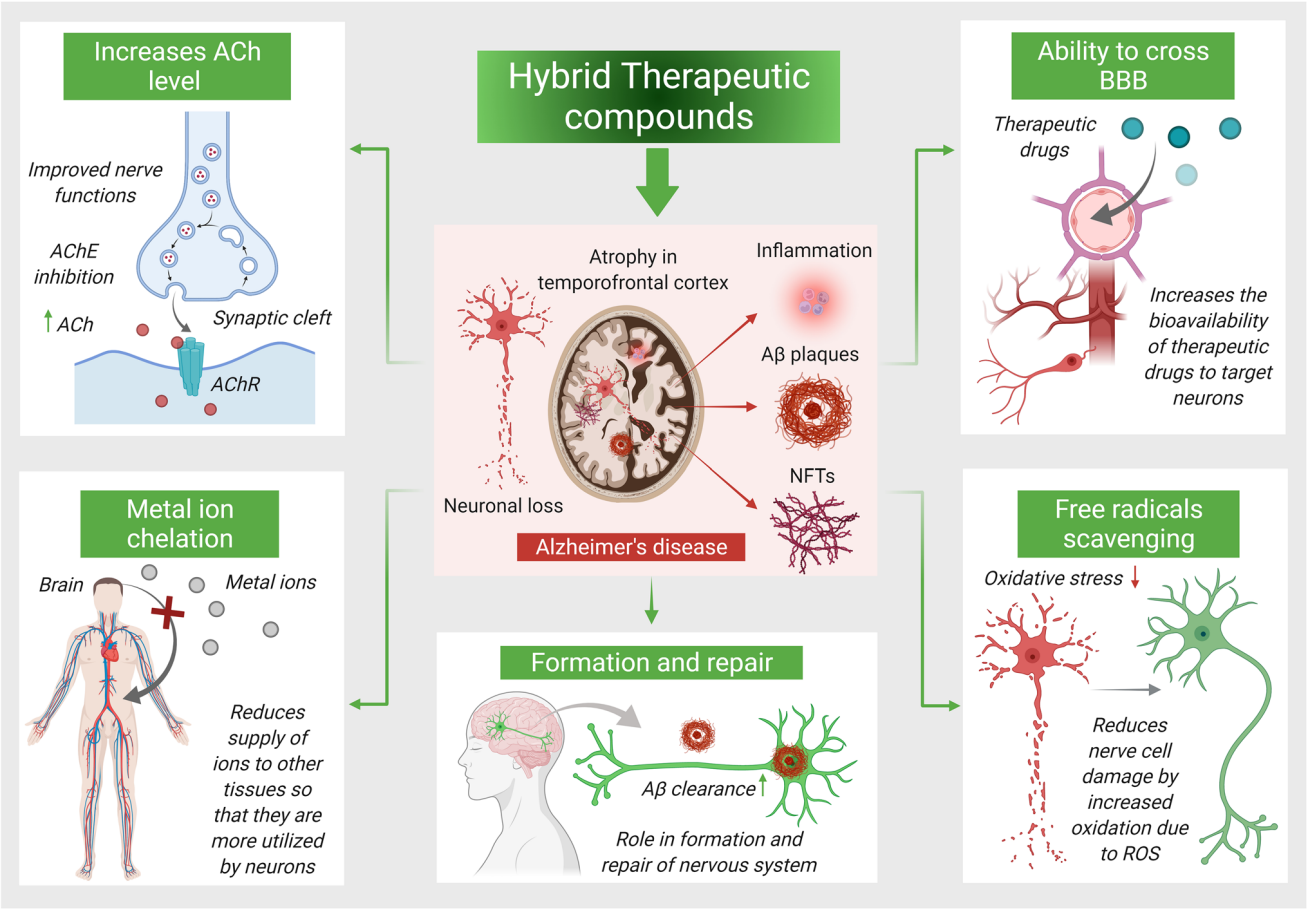
Hybrid multitargeted therapeutic compounds against multifactorial character of AD

There are numerous options for designing hybrid therapeutic compounds, mostly possible due to their common structural properties at molecular level, with better targeting to the specific molecular sites. Hybrid derivatives can combine a variety of chemical components from different resources (organic moieties, peptides, prodrugs, and others). These hybrid compounds have shown high affinity against a specific disease by exerting complementary actions such as dual mode of action, carrier or barrier crossing attributes, cellular subclass targeting, or other complementary actions (Fig. 2) [43].

### Various Hybrid Therapeutic Compounds Targeting AD

**Fig. 3:**
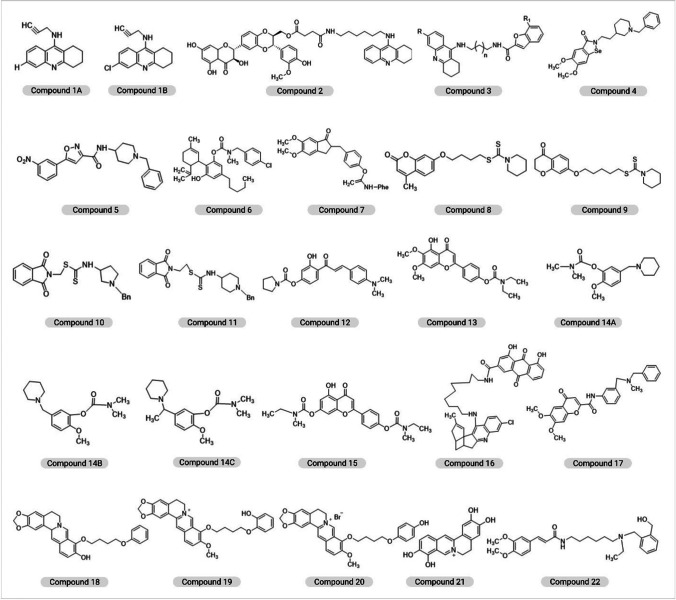
The chemical structure of hybrid compounds along with their serial number

Lack of disease-altering medications to treat AD has generated a flurry of research and the numerous studies are under-going for the development of multi-target therapies in order to tackle the complications occurring during AD progression [44]. Tacrine (TAC), donepezil (DP), and rivastigmine (RSM) are some of the most widely explored drugs that can be conjugated or hybridized to target additional AD targets. The chemical structure of hybrid compounds along with their serial number is depicted in Fig. 3.

## Tacrine-Conjugated Hybrids

Acetylcholine (ACh) is a neurotransmitter that encodes memory, and a reduced Ach activity is indicative of the severity of AD [45–47]. TAC is a FDA-approved acetylcholinesterase inhibitor (AChEI) for the management of AD [48]. Even though TAC possesses various adverse effects, it is directed to patients suffering from AD for its enhanced ability to block AChE [49]. Moreover, to overcome the hepatotoxicity and neurotoxicity issues associated with TAC, its hybrid compounds are synthesized.

The first TAC-based hybrids were utilized via THA-propargylamine (*compound 1*), a compound containing a propargylamine group for neuroprotective effects. The THA moiety and the propargylamine in combinations were used as a novel heterodimers for enhancing the AChEI activity and limiting the hepatotoxicity, making it as a potential candidate for treating AD [50]. Chen et al. designed and prepared another hybrid (*compound 2*) combining THA and silibinin via silibinin (silymarin complex) having anti-inflammatory, anti-cancer, and neuroprotective and hepatoprotective properties. *Compound 2* displayed potent cholinesterase inhibitory activity but appears less potent AChEI than THA, though it was able to downregulate AchE activity with neuroprotective effects and reduced hepatotoxicity, both in vitro and in vivo [51]. Tang et al. developed an avant-garde hybrid of oxoisoaporphine and THA, possessing Aβ aggregation and anticholinergic activity for self or induced AChEI activity. Oxoisoaporphine alkaloids isolated from the rhizome of *Menispermum dauricum* showed high AChEI inhibitory activity and better selectivity of AChE over BChE. An amino alkyl ether comprised of a secondary amine and amide bond was connected using spacer, THA fragment with the oxoisoaporphine moiety [52]. Eriksen et al. synthesized novel multifunctional drug candidates by combining THA with flurbiprofen, a non-steroidal anti-inflammatory drug (NSAID) reduced the production of Aβ_40-42_ via flurbiprofen that targets the complex, γ-secretase, responsible for the formation of Aβ from APP [53]. Fancellu et al. synthesized another novel hybrid by coupling TAC to benzofuran (BF) derivatives. The BF derivatives imparts the conjugate molecules have ability to inhibit AChE and Aβ peptide aggregation. Moreover, it improved the ability to chelate metals ( Cu^2+^ and Fe^2+^) and associated extra antioxidant activity, for the hybrids with hydroxyl substitution. The novel TAC-BF hybrids (*compound 3*) enhanced AChEI activity and significantly prevented self- and Cu-mediated Aβ aggregation which is dependent on linker size and substituent groups of each moiety along with neuroprotective effects [54].

Ferulic acid (FA), an antioxidant of natural origin found in esterified forms with saccharides, sterols, and lignin, is taken up via the gastrointestinal tract, metabolized by the liver. Moreover, it makes THA-FA derivative moderately antioxidant and a potent reversible, non-competitive AChEIs [55]. Rodriguez-Franco et al. synthesized a novel THA melatonin derivative as a potential multi-targeting drug ligand that could be utilized in the treatment of AD. Melatonin is a pineal neurohormone that not only possesses potent antioxidant activities and aids in scavenging ROS but also provide protection against Aβ-triggered apoptosis in microglial cells [56]. Furthermore, antioxidant capacities were also tested in an oxygen radical absorbance capacity assay using fluorescein. The compound developed by the group act as a strong inhibitor of human AChE and showed high oxygen radical absorbance capacity. Thus, various TAC-based hybrids exhibited therapeutic activities with multiple relevant properties and could be considered potential multitargeting anti-AD agents.

## Donepezil-Based Hybrids

The AChEI boosts brain cholinergic neurotransmission by increasing endogenous ACh levels. DP (2-((1-benzylpiperidin-4-yl)methyl)-5,6-dimethoxy-2,3-dihydro-1H-inden-1-one) is one of the most effective and well-known FDA-approved AChEI [57]. DP constitutes of dimethoxy indanone and a methylene linker that connects it to N-benzylpiperidine. It demonstrates AChEI activity, anti-Aβ aggregation, and antioxidant as well as metal chelating activities [58]. Luo et al. investigated a new class of chemicals by combining DP, the cholinesterase inhibitor with ebselen, an antioxidant to generate multi-target-directed ligands (MTDLs) against AD. An AChEI, *compound 4* (*IC*_50_ = 0.042 μM for *Electrophorus electricus* AChE and *IC*_50_ = 0.097 μM for hAChE), has been discovered to be a strong BChE inhibitor (*IC*_50_ = 1.586 μM) having H_2_O_2_ and peroxynitrite scavenging activity, glutathione peroxidase-like activity (*ν*_*o*_ = 123.5 μM min^−1^), and found to be a substrate of class *TrxR* with no issues. *Compound 4* potentially permeated the CNS, showed in an in vitro BBB model [44]. Arce et al. used a linker that is biocompatible, named L-glutamic acid, a novel multifunctional DP-based derivative for the treatment of AD. The distance between the αNH and the γCO_2_H makes L-glutamic acid a good choice to permit simultaneous interaction between two primary sites, catalytic active site (CAS) and peripheral anionic site (PAS). Cholinesterase inhibitors significantly displace propidium iodide from AChE for inhibition of amyloid aggregation and penetrate brain and improves cell viability, in vitro. They also inhibit human AChE and BChE, protect neurons from mitochondrial free radical damage, and are likely to enter the CNS through passive diffusion [59]. In another study Monjas et al. elucidated a new DP-based L- and D-glutamic acid derivative exhibiting potential neuroprotective effects that combat oxidative stress caused by a combination of rotenone and oligomycin A or oxygen and glucose deficiency [60]. In a recent investigation, Saeedi et al. synthesized *N*(*1benzylpiperidin4yl*)*5 aryl isoxazole 3 carboxamide* derivatives and tested it against AChE and BChE. Based on the in vitro results, *compound 5* appears most potent inhibitor of AChE (*IC*_50_ = 16.07 μM) and BChE (*IC*_50_ = 15.16 μM) and exhibited selective anti-AChE activity (*IC*_50_ = 23.63 M). Thus, aryl isoxazole benzylpiperidine-based compounds could be considered potential agents for the treatment of AD [61]. A new class of multifunctional hybrids has been developed and tested by Piemontese et al. that could be potential agents for AD. These are based on the structure of the DP drug that imitates the structure of DNP by conjugating a benzylpiperidine/benzylpiperazine fraction with bioactive heterocyclics (benzimidazole or benzofuran). These endowed the hybrids with additional features like inhibition of Aβ peptide aggregation, antioxidant activity, and metal chelation. Altogether, the compounds displayed the well ability to inhibit AChE (*IC*_50_ = 4.0–30.0 μM) [62]. Camps et al. created some DP-TAC hybrids that link with AChE’s active, peripheral, and mid gorge binding sites all at the same time. AChE, BChE, and Aβ-aggregation influenced by AChE were showed inhibited by these hybrids. The hybrids of DP-TAC were made by joining 6 chloro-TAC with the indanone fraction of DP and they appear more potent than their parent compounds in inhibiting hAChE [63].

## Carbamate-Conjugated Hybrids

Carbamates (CM) are an important compound due to their superior chemical stability owing to the carbamic group, and ability to increase the permeability of biological membranes. It serves as a crucial structural motif in several licensed medicines and pro-drugs. Recently, studies reported significant application in their usage for developing agents with potential to target their CM moiety. During the process of enzymatic inhibition carbamic group binds to the CAS triad in the active site of AChE to release the phenolic counterpart which exhibits therapeutic potential against AD attributed to the antioxidant mediated neuroprotective activities against AD [64].

Jiang and colleagues investigated the binding ability of a CM fragment to the active site of BChE through structural splicing approach which is based on docking. Based on the aforementioned approach, seventeen new compounds were generated via structural re-assembly but *compound 6* was the only compound that exhibited a highly selective BChE inhibitory activity. It possesses the ability to penetrate BBB and offers harmless, neurological protection, antioxidant, and pseudo-irreversible BChE inhibition. In-vivo results revealed that *compound 6* significantly reduced scopolamine-induced cognitive impairment and recuperated the Aβ_1-42_ (icv)-impaired cerebral function. Thus, *compound 6* exhibited better efficacy than DP with a decent anti-amyloidogenic effect, and so it could be potentially used as a potential therapeutic agent in AD [65]. In another investigation, Gargari et al. designed a new set of indanone-CM hybrids based on the pharmacophore hybridization technique. These compounds were investigated in order to inhibit AChE and BChE, and it was observed that the *compound 7* displayed the highest AChEI activities through reversible partial non-competitive inhibition and also act as a potent Aβ_1-40_ aggregation inhibitor [66]. Jiang et al. designed coumarin-dithiocarbamate hybrid as versatile compound for AD. *Compound 8* is a type of variegated inhibitor that concomitantly interacts with the CAS and PAS in order to hinder AChE and exhibited potent inhibition of Aβ aggregation. Moreover, it possesses the ability to chelate specific metals, better BBB permeability, and reduced neurotoxicity, both in vitro and in vivo and thus may be promising compound in the quest for drugs against AD [67]. Jiang et al. developed a group of hybrids acting as unique multifunctional AChE inhibitors by attaching chromanone to dithiocarbamate moieties using flexible linkers to inhibit self- and AChE-induced aggregation of Aβ. Moreover, the potential to infiltrate the BBB and reduce neurotoxicity in neural cells highlighted the importance of *compound 9* as a potential drug against AD [68]. In another investigation, inhibitory potentials of a novel set of phthalimide-dithiocarbamate hybrids were examined by Asadi et al. against AChE and BChE in vitro. The anti-cholinesterase activity of *compounds 10 and 11* could also possess properties similar to drugs and cross the BBB, thereby demonstrated the capability to be used in AD [69].

### Rivastigmine-Based Hybrids

Rivastigmine (RSM) has received approval from the FDA for utilization as a transdermal patch for the management of AD at a mild, moderate, and severe stage. It has both AChE and BChE inhibitory activity. Moreover, with severity of the disease stages, a surge in the BChE levels in the temporal cortex and hippocampus is observed in people with AD, however AChE activity decreases. Therefore, an inhibitor which exhibit effect on both will increase ACh levels drastically. Considering mechanism of action, it behaves similarly to ACh as it attaches to both the anionic and esteratic sites of AChE. Instead of dissociating immediately post hydrolysis, which happens in ACh, RSM gets hydrolyzed leaving the esteratic site of AChE carbamylated for a period of time, thereby inhibiting the enzyme. It is also known to inhibit the G1 enzymatic form of AChE, which is typically prevalent in the brains of AD patients [70].

RSM-caffeic acid and RSM-FA hybrids were developed, synthesized, and assessed in vitro as potential multifunctional medicines for AD by Chen et al. The newly produced hybrids showed antioxidant and neuroprotection activities, and also upregulated ChE inhibition but simultaneously decreased the aggregation of Aβ [71]. In another study, Xiao et al. developed and examined therapeutic potential of 4′-amino chalcone-RSM hybrids as multifunctional medicines in AD therapy. *Compound 12* inhibited AChE and substantial anti-oxidative activity with reduced hepatotoxicity and permeated through the BBB in vitro. Furthermore, it also exhibited self-induced Aβ_1-42_ anti-aggregation properties as well as inhibited Aβ_1-42_ aggregation, induced due to Cu^2+^, by selectively acting as a MAO-B inhibitor and metal chelator [72]. Sang et al. reported that scutellarin-RSM hybrids exhibited cholinergic, antioxidant, bio-metal chelating, and neuroprotective properties against AD. Certain types of scutellarein carbamate derivatives were made using MDLS and the *compound 13* exhibited dual inhibition of AChE and BChE, bio metal-chelating properties, antioxidant properties, and also provided neuroprotection against cell injury induced by H_2_O_2_ using in vitro murine model [73]. Li et al. created a novel series of 2-methoxy-phenyl dimethyl-carbamate derivatives to evaluate site-activated MTDLs on the basis of curcumin and RSM. *Compound 14A*, *compound 14B*, and *compound 14C* exhibited dual AChE and BChE inhibition with IC_50_ value less than a micromole against self-aggregation of Aβ. Among the compounds developed, *compound 14B* showed the maximum competency for AChE inhibition which was about 20-times more when compared to that of RSM and the hydrolysate of *14B* exhibited the potential for Cu^2+^ and Fe^3+^ chelation in vitro [74]. A multimodal drug, Ladostigil (TV3326) [(N-propargyl-(3R) aminoindan-5yl)-ethyl methyl carbamate], was combined with rasagiline, an anti-Parkinsonian drug, and specific MAO-B inhibitor and showed that hybrid compounds potentially inhibited the ChE activity of RSM [75]. Sang et al. developed novel apigenin-RSM hybrid via MTDLs approach owing to antioxidant potency and a reversible huAChE and huBChE inhibitory potential using in vitro model. *Compound 15* also acts as a selective metal chelator, exhibited self-mediated and Cu^2+^-mediated Aβ_1-42_ anti-aggregation property, causing suppression of huAChE-mediated induced Aβ_1-40_ aggregation. Moreover, *compound 15* displayed neuroprotective and hepatoprotective activity, favorable for BBB infiltration in vitro and drug-like properties, thus could be used as a potential agent to target numerous AD-associated factors [76].

## Physostigmine-Conjugated Hybrids

Physostigmine (PSM) or Eserine, originally isolated from Calabar beans has been found useful in AD due to its inhibitory actions on AChE. Potent novel analogs of PSM showing AChE inhibitory property (IC50 = 0.14 nM) were developed wherein alkoxy groups were utilized to attach N-methyl-N-(3-carbamoyloxyphenyl) methylamino derivatives with the tertiary amino nitrogen of parent PSM [77]. In addition to AChE inhibitory actions, they also showed the potential to improve memory [78]. Tolserine and phenserine are some of the effective ChE inhibitor derivatives.

A chemical derived from PSM, phenserine ((-)-eseroline phenyl carbamate) appears an effective, non-competitive, long-acting, and selective inhibitor of AChE [79]. Klein et al*.* reported that phenserine inhibits AChE and reduces the production of APP through interaction with a regulatory element in the 5′-UTR region APP gene to reduce the efficiency with which APP mRNA translates into a protein, Aβ. This process is mediated by an interaction with Fe^3+^ or an Fe^3+^-responsive element and it impact the formation of Aβ in vivo and in vitro [80].

The only difference between tolserine and phenserine is the 2-methyl substitution on the phenyl carbamoyl molecule. Mehta et al. showed that tolserine was 200 times more selective for hAChE than BChE [81]. Tolserine showed the *IC*_50_ value of 0.01 µM against AChE in human erythrocytes [82]. Eu et al. found the *IC*_50_ value using Ellman Technique was 0.0103 µM against the hAChE in RBCs [83]. It has also found that the potency of tolserine to human AChE is much higher than phenserine or PSM [84].

## Galantamine-Based Hybrids

Galantamine (GAL), a tertiary alkaloid of natural origin acts as a reversible, competitive inhibitor of the AChE enzyme. It is approved by FDA in year 2001 for use in the management of mild to moderate cognitive impairment in AD patients. GAL potentially inhibits the AChE in the synapse, hence enhances the function and signaling of the cholinergic neurons. GAL found to influence the progression of the disease and facilitates retaining the functions of the cholinergic neurons [85]. The efficacy of GAL is often compromised by its adverse effects and limited success in preventing the worsening of a patient’s condition [86].

Curcumin (CU), a natural polyphenolic compound chemically known as diferuloyl methane has garnered attention for its therapeutic potential in neurodegenerative diseases including AD. It possesses numerous benefits such as delayed degradation of neurons, decreased Aβ plaques, metal-chelation, antioxidant, and reduced microglia formation and anti-inflammation, which makes it as a potential candidate in improving cognitive features and curtailing pathogenesis in AD [87]. Stavrakov et al. developed a hybrid compound composed of GAL core combined with CU fragments to obtain the synergistic effect. Moreover, CU can attach to the oligomers of Aβ and prevent plaque formation. A GAL-CU hybrid was synthesized as a new non-toxic AChEI with increased antioxidant activity that served as a lead compound and explored for therapeuric potential in AD [88, 89]. GAL attaches properly over the AChE binding gorge but the size does not seem apt enough for occupying it entirely. The peptide of Aβ attaches with the PAS at the entryway of the binding site of AChE and induces amyloid plaque production; however, PAS inhibition thwarts Aβ aggregation induced by AChE. Stavrakov et al. investigated the potential of a set of GAL-camphane hybrids in AD which act as AChEIs. Camphane is a large component that adheres to the wide opening of the gorge that is made with varying lengths of linkers attached to it, therefore the compound could attach to the gorge. The camphene fragments belonging to the best binders attach in the same position, closely located to the PAS and the site where Ω-loop of Aβ attaches to AChE. These hybrids penetrated through the BBB via passive diffusion and appears devoid of neurotoxicity, even at the inhibitory concentrations [90].

## Rhein/Huprine-Conjugated Hybrids

According to Viayna et al. huprine and rhein were linked via a distinct length alkyl or aryl alkyl chain (5 to 11 carbon atoms). Both hAChE and hBChE were inhibited by the synthesized huprine-rhein hybrids, with the value of *IC*_50_ in the nanomolar and sub-micromolar to low micromolar ranges, respectively. The novel rhein-huprine hybrid compounds were physiologically assessed against AChE, BChE, dual A*β*_42_, BACE-1, and anti-tau accumulating properties in *E. coli*. The hybrids comprised of a rhein and a huprine Y structure joined through penta- to un decamethylene or 1,4-phenylene-bis(methylene) linkers. *Compound 16* exhibited disease-modifying anti-Alzheimer’s therapeutic candidate with hAChE (*IC*_50_ = 2.39 nM), hBChE (*IC*_50_ = 513 nM), BACE-1 (*IC*_50_ = 80 nM), and 43% accumulation of Aβ_42_ at 10 μM. In vivo results showed that, it also inhibited Aβ-induced synaptic loss of protein and lowering of central Aβ in APP-PS1 mice [91]. Pérez-Areales et al. developed similar type of multitarget hybrid molecules comprised of rhein with a unit of the effective AChEI, huprine Y. In vitro studies revealed that the hybrid compound exhibited an intriguing multi-targeting property, including cholinergic activity via inhibition of hAChE (*IC*_50_ = 3.60 nM); hBChE (*IC*_50_ = 620 nM), and anti-aggregating activity for Aβ_42_ and tau (48% and 30% inhibition at 10 μM, respectively), in a cell-based assay utilizing *E. coli*. This compound also inhibited human BACE-1 (*IC*_50_ = 120 nM), the enzyme represent rate-determining stage of Aβ production from APP, leading to reduced level of Aβ in a transgenic murine strain of AD. The 3′-basicity huprine component is crucial for the inhibition of AChE and BACE-1. The huprine moiety of this hybrid reacts with the catalytic site of both AChE and BACE-1 when protonated at physiological pH. It allows cation–π interactions with Trp86’s indole ring and Tyr337’s benzene ring. It also forms a salt bridge with the catalytic dyad’s Asp32 and Asp228 residues in BACE-1 [92]. Furthermore, the role of different hybrid therapeutic compounds for the management of AD is discussed in Table 1.

**Table 1:** Role of different hybrid compounds against AD

Hybrid compound	AChE inhibitor	β-amyloid antiaggregation	Antioxidant	Other activities	*IC*_50_ value(for AChE)	Clinical study	References
N-(prop-2-yn-1-yl)-1,2,3,4-tetrahydroacridin-9-amine (Cpd1A)	✔□			BChE inhibitory activities	51.3 nM	In vitro	[50]
6-Chloro-N-(prop-2-yn-1-yl)-1,2,3,4-tetrahydroacridin-9- amine (Cpd1B)	✔□			BChE inhibitory activities	11.2 nM	In vitro	[50]
Mixture of silibinin hemisuccinate and 6- aminohexamethylene tacrine (N1 -(1,2,3,4-tetrahydroacridin-9- yl)hexane-1,6-diamine)Cpd2	✔□		✔□	BChE inhibitory activities	53.9 nM	In vivo, In vitro	[51]
Oxoisoaporphine-tacrine	✔□	✔□		NA	nM range (41–57 nM)	In vitro	[52]
Tacrine–benzofuran hybrid Cpd 3	✔□	✔□	✔□	Metal chelation activity	38.6 nM	In vitro	[54]
Tacrine-melatonin hybrid	✔□		✔□	Able to cross BBB	0.008 nM(40 000-fold more potent than tacrine)	In vitro	[56]
Tacrine-ferulic acid hybrid	✔□	✔□	✔□	Inhibition of the PAS of AChE BChE inhibitory activities	4.4 nM	In vitro	[55]
Donepezil and Ebselen hybrid Cpd 4	✔□	✔□	✔□	Butyrylcholinesterase inhibitor (*IC*50 = 1.586 μM), peroxynitrite scavenging activity and glutathione peroxidase-like activity (*ν*0 = 123.5 μM min–1)	0.097 μM	In vitro	[44]
DNP based L-glutamic acid hybrid	✔□	✔□	✔□	BChE inhibitory activities, BBB permeation ability	0.10–0.53 μM	In vitro	[59]
N-Cbz-L-Glu(OEt)-[NH-2-(1-benzylpiperidin-4-yl)ethyl] (L-3)	✔□			Protected rat hippocampal slices against oxygen–glucose deprivation, becoming promising anti-Alzheimer's and anti-stroke lead compounds	4.99 µM	In vitro	[60]
N-Cbz-L-Glu(OEt)-[NH-2-(1-benzylpiperidin-4-yl)ethyl] (L-1)	✔□			Blocks the voltage-dependent calcium channels	0.53 µM	In vitro	[60]
Donepezil-N(1benzylpiperidin4yl)5 aryl isoxazole 3 carboxamide derivative	✔□	✔□		BChE inhibitory activities	16.07 μM	In vitro	[61]
Donepezil-tacrine hybrid	✔□	✔□		BChE inhibition	Subnanomolar or low nanomolar range	In vitro and in silico	[63]
Donepezil-Benzylpiperidine hybrid	✔□	✔□	✔□	Tau hyperphosphorylation inhibition, metal chelation activity	4.0–30.0 μM	In silico	[62]
Carbamate derivative Cpd 6	✔□	✔□	✔□	Penetrates BBB, offers benign safety, neuroprotection, and pseudo-irreversible BChE inhibition	5.3 nM (for BChE)	In vivo and in silico	[65]
Indanone–carbamate hybrid Cpd 7	✔□	✔□		NA	4.64 μM	In vitro and in silico	[66]
Coumarin-dithiocarbamate hybrid Cpd 8	✔□	✔□		Metal-chelating ability, good BBB permeability and low toxicity on SH-SY5Y neuroblastoma cell	0.027 μM	In vitro and in vivo	[67]
Chromanone-dithiocarbamate hybrid Cpd 9	✔□	✔□		Ability to penetrate the BBB and low neurotoxicity in SH-SY5Y cells	0.10 μM	In vitro, in vivo and in silico	[68]
Phthalimide-dithiocarbamate hybrid Cpd 10	✔□			Anti BChE activity, possesses drug-like properties and able to cross the BBB	4.6 μM	In vitro and in silico	[69]
4′-aminochalcone-revastigmine hybrid	✔□	✔□	✔□	Selective monoamine oxidase B inhibitor and a selective biometal chelator	4.91 μM	In vitro	[72]
Scutellarein carbamate derivative Cpd 13	✔□		✔□	Bio-metal chelating and neuroprotective properties	0.57 μM	In vitro	[73]
4′-aminochalcone-revastigmine hybrid Cpd 12	✔□	✔□	✔□	Selective monoamine oxidase B inhibitor (IC50 = 0.29 μM) and a selective biometal chelator	4.91 μM	In vitro	[72]
2-methoxy-phenyl dimethyl-carbamate derivative Cpd 14B	✔□	✔□		Potent ABTS^**.**+^ scavenging and moderate copper ion chelating activity	0.097 μM	In vitro	[74]
Apigenin-rivastigmine hybrid Cpd 15	✔□	✔□	✔□	Remarkable dyskinesia recovery rate and response efficiency	6.8 μM	In vitro and in vivo	[76]
Phenserine	✔□	✔□		NA	22 nM	In vitro and in vivo	[80]
Tolserine	✔□			NA	0.01 µM	In vivo	[82]
Galantamine (GAL) and curcumin (CU) hybrid	✔□		✔□	NA	7.91 to 52.53 µM	In vitro	[88, 89][87]
Galantamine-camphane hybrid	✔□	✔□		NA	0.0029–0.0099 µM	In silico	[90]
Cpd 16	✔□	✔□		Tau hyperphosphorylation inhibition	2.39 nM	In vivo	[91]
Naphthyridine- and thienopyridine-based rhein-huprine hybrids	✔□	✔□	✔□	Tau hyperphosphorylation inhibition	3.60 nM	In vitro	[92]

## Novel Hybrid Therapeutic Compounds Targeting AD

Along with the aforementioned hybrid therapeutic complexes, also plenty of new hybrid compounds have been synthesized exhibiting potential therapeutic efficacy in the management of AD development (Table 2). The activities of these novel hybrid therapeutic compounds have been discussed in succeeding paragraphs.

**Table 2:** Novel hybrid therapeutic compounds against AD

Hybrid compound	AChE inhibitor		β-amyloid antiaggregation	Antioxidant	Other activities	IC_50_ value	Clinical study	References
Amentoflavone			✔□		NA	0.26 µM	In vitro	[93]
*N*-(3-((Benzyl(methyl)amino)methyl)phenyl)-6,7-dimethoxy-4-oxo-4*H*-chromene-2-carboxamide Cpd 17		✔□			NA	4.5 µM	In vitro and in silico	[94]
Cpd18, berberine linked to phenol by 4-carbon spacers		✔□			NA	0.097 µM	In vitro and in silico	[99]
Berberine-pyrocatechol hybrid (compound 19)		✔□			NA	0.123 µM	In vitro	[100]
Berberine-hydroquinone hybrid (compound 20)		✔□	✔□	✔□	NA	0.460 μM	In vitro	[100]
Ber-D Cpd 21				✔□	Cu chelation, reduces cellulo multifaceted toxicity in AD	NA	In vitro and in silico	[101]
Bis(9)-( −)-Meptazinol (B9M)		✔□	✔□		NA	3.9 nM	In vitro	[102]
Ferulic acid-memoquin hybrids, Cpd 22		✔□	✔□	✔□	Can cross the BBB	3.2 μM	In vitro	[103]

The biflavonoids have been tested by Choi et al. and the result showed that amentoflavone (*IC*_50_ = 0.26 µM) significantly inhibited Aβ_1-42_ fibrillization most efficiently. According to the structure–activity relationship analysis, the –OH groups of biflavonoid molecules are crucial in their chemical interaction with the active course of Aβ_1-42_ fibrillization. Amentoflavone interrupted the granular shape of formed Aβ_1-42_ threads, causing them to form nebulous Aβ_1-42_ aggregates. Many such consequences revealed that amentoflavone seems to have the most potent action across equally Aβ_1-42_ fibrillization suppression and fully developed Aβ_1-42_ fibril disaggregation, indicating that it could be used to treat the disease [93]. In another study, Estrada-Valencia et al. performed a funnel-type screening of many CNS-permeable flavonoid-DBMA hybrids and found that *compound 17* showed the MTD-profile in human AChE, hLOX-5, hBACE-1, and σ1R (*IC*_50_ (hAChE) = 4.5 µM; *IC*_50_ (hLOX-5) = 30 µM; *IC*_50_ (hBACE-1) = 6.7 µM; and *IC*_50_ (σ1R) = 0.5 µM). *Compound 17*, which could aid neurological rejuvenation and actually hinder neurodegeneration in AD was developed through molecular mechanics simulations to get an effective agent with protein relationship [94].

Berberine (BB), an isoquinoline alkaloid is one of the important ingradient of many conventional Chinese medicines which are used in the treatment of illnesses including inflammatory and diarrheal diseases, high blood pressure, and carcinomas [95–97]. BB has been shown to interfere with AD pathogenic processes through lowering the levels of Aβ by impeding activity of secretase enzymes in the APP pathway, alleviating astrocytosis, mitigating oxidative stress, and hindering neurological exaggeration [98]. Huang et al. developed and tested the inhibitory activity of three courses of its derivatives against AChE. Majority of them have been found powerful AChE suppressors, with *IC*_50_ values in the micromolar range. The far more powerful suppressor, *compound 18*, BB has been connected to phenol by 4-carbon inserts, hindered AChE with an *IC*_50_ of 0.097 µM. The derivatives prompted a mixed form of inhibitory activity and engaged among both CAS and PAS, following the kinetic model [99]. Jiang et al. successfully synthesized a sequence of hybrid compounds through reacting BB to Benzene-1,2-diol, N-Acetyl-5-Methoxytryptamine, and FA, agent showed potential for AD, was modified as BB-pyrocatechol hybrid (*compound 19*) appear more efficient in inhibiting AChE than the parent BB (*IC*_50_: 0.123 vs 0.374 μM). Not only that, it was also figured out that the berberine-hydroquinone hybrid (*compound 20*) showed greater antioxidant activities and was also able to inhibit AChE (*IC*_50_ of 0.460 μM), with an additional feature of inhibiting Aβ aggregation. The location of phenolic hydroxyl over the benzene ring in *compound 20*, a hydroquinone-BB derivative, tends to affect the chemical’s potential to block Aβ aggregation, similar to its critical role in inhibiting AChE and BChE [100]. Recently, Rajasekhar et al. reported a multifunctional natural substance derived from BB (Ber-D) that reduces cellulo multifaceted toxicity in AD. The structural characteristics of polyphenol Ber-D (*compound 21*) have also presented to its effective Cu^2+^ chelation along with redox cycle arresting properties, preventing the formation of ROS. In silico simulations show that Ber-D suppresses both metal-dependent and metal-independent Aβ aggregation and also prevented mitochondrial malfunction and neuronal toxicity, leading to early apoptosis. Ber-D can be a promising therapeutic option in mitigating complex Aβ toxicity in AD owing to these significant multifunctional properties [101].

Shi et al. examined the effect of Bis(9)-( −)-Meptazinol (B9M), a unique putative multifunctional binding AChEI, on memory and cognitive abilities in the APP/PS1 mouse model of AD. In the Morris water maze test, nest-building test, and new object recognition experiment, B9M therapy markedly enhanced the cognitive ability of APP/PS1 transgenic mice. In early investigations, B9M was banded over CAS and PAS of AChE and showed an efficient AChE inhibitory activity (*IC*_50_ = 3.9 nM). B9M also inhibited the AChE-triggered Aβ accumulation, suggesting that it could be useful to treat AD [102].

**Fig. 4:**
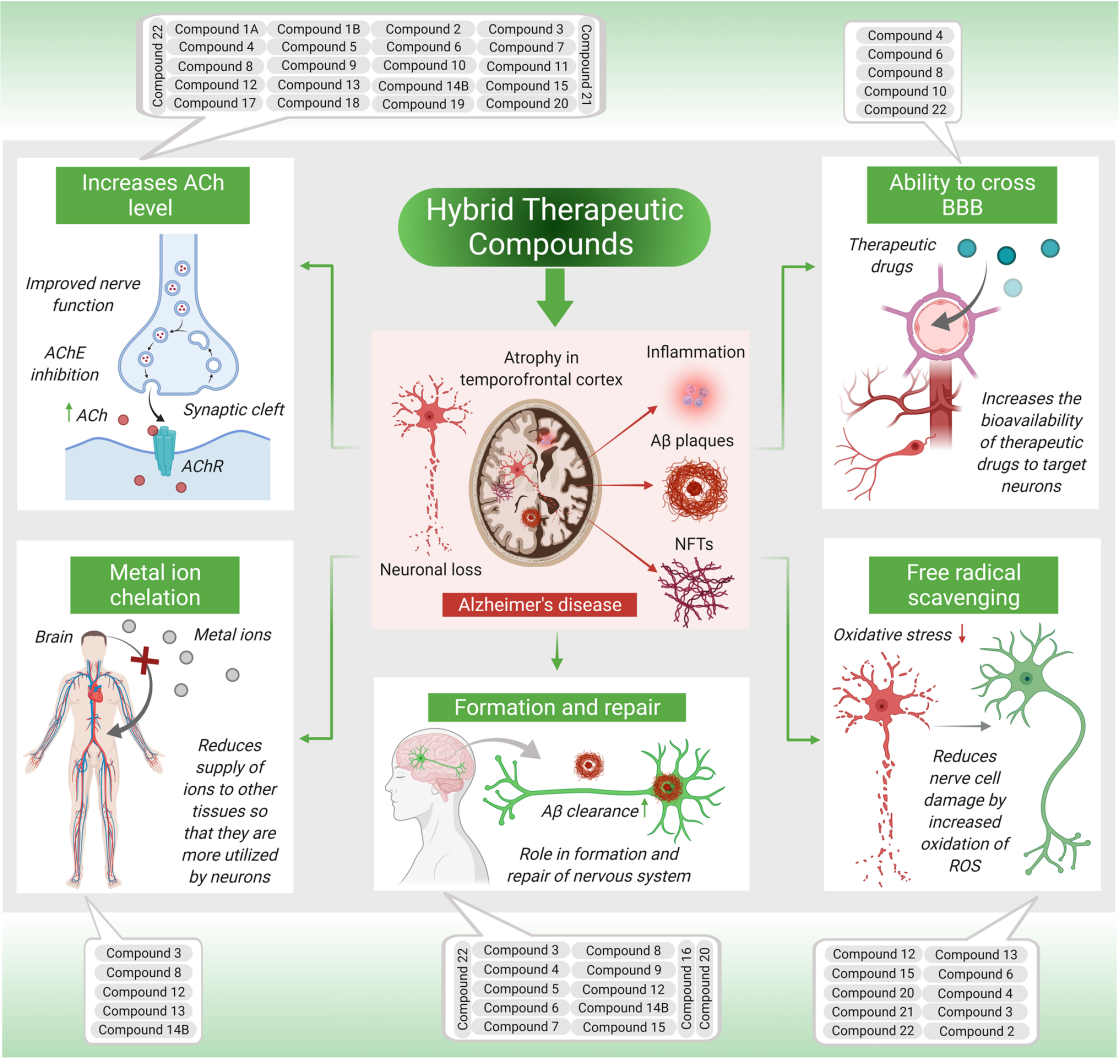
Illustration shows specific role of each hybrid therapeutic compound against AD

Pan et al. synthesized, and evaluated a new class of FA-memoquin hybrids as multifaceted medicines for AD. The in vitro experiments revealed that the majority of the compounds exerted a significant inhibitory effect on AChE (*IC*_50_ of 3.2–34.7 μM). Especially, *compound 22* showed significant and found superior in inhibiting AChE (*IC*_50_ = 3.2 μM) and the accumulation of Aβ_1-42_ (30.8%). It was also an exceptional antioxidant and neuroprotectant. Additionally, *compound 22* could cross the BBB in vitro while demonstrating that the benzylamines group exerted important outcome on the antagonistic effect of AChE, usually, the -CH_3_ group at the 2′-position of benzylamine exhibited inhibition, and the substituted groups possessing an N-ethyl group indicated improved AChE inhibition than that with an N-methyl group. Furthermore, the inhibitory activity was enhanced with the extension of the methylene linker [103]. Furthermore, as discussed in aforementioned sections, the specific role of each hybrid therapeutic compound against AD is illustrated in Fig. 4.

## Conclusion and Future Perspectives

In summary, the pursuit of an effective and safe medications for AD continue to be a challenge for  medicinal chemists and neuroscientists. Since the pathogenesis of AD includes multiple characteristics including dysregulation of diverse molecular targets and biochemical pathways, therefore achieving therapeutic benefits from a single agent and single target remains a challenge with potential therapeutic agents. The use of highly selective drugs targeting AD has made the therapeutic strategies ineffective, leaving the patients with no other options but to use multiple drugs combinations for regulating a diverse series of symptoms associated with the progression of AD. Therefore, a great deal of recent research aimed to the discovery of novel bioactive hybrid compounds targeted towards the unique, diverse, and prospective pathogenic hallmarks of AD. An effective therapy for AD may rely on if the novel developed agent exert the potential for interaction and regulate different molecular targets and simultaneously block or activate  numerous biochemically linked pathogenic pathways. Moreover, the reported studies have provided adequate evidence for the favourable outcome of poly-pharmacology in controlling AD pathogenesis. This is more compelling particularly regarding the utility of a single drug showing different modes of action, with reduced toxic effects, metabolic overloads, and drug-drug interactions. In this field, numerous compounds with multi-target profiles have been discovered, and many of them have shown intriguing and promising pharmacological characteristics, making them prospective therapeutic candidates. The hybrid therapeutic complexes are mainly developed with the specific combination of two bioactive pharmacophores to produce homo- and heterodimers with an improved affinity, therapeutic efficacy, biological profile, and additional complementary effects. Thus, many disease-modifying hybrids could be potentially developed into next-generation medicines for AD. The method of designing hybrid molecules has numerous advantages over conventional multitarget drug development methods. In comparison to in silico high throughput screening, hybrid molecules generate quicker results and are also less expensive than fragment-based drug development. Designing hybrid multitargeted therapeutic compounds is thus a prospective approach in developing an effective treatment for AD. Nevertheless, several issues must be addressed, and additional researches should be conducted to develop hybrid therapeutic compounds for clinical usage while keeping other off-target adverse effects in mind.

## Data Availability

Not applicable.

## Abbreviations

Aβ: β-amyloid
Ach: Acetylcholine
AChE: Acetylcholinesterase
AChEIs: Acetylcholinesterase inhibitors
AD: Alzheimer’s disease
ADME: Absorption, distribution, metabolism, and excretion
APP: Amyloid precursor protein
B9M: Bis(9)-( −)-meptazinol
BACE-1: Beta-secretase 1
BBB: Blood-brain barrier
BChE: Butyrylcholinesterase
BF: Benzofuran
CAM: Camphane
CAS: Catalytic active site
ChEs: Cholinesterases
CNS: Central nervous system
CSF: Cerebrospinal fluid
CU: Curcumin
DBMA: N,N-dibenzyl(N-methyl)amine
DNA: Deoxyribonucleic acid
FA: Ferulic acid
FDA: Food & Drug Administration
GAL: Galantamine
hAChE/huAChE: Human acetylcholinesterase
IC_50_: Half-maximal inhibitory concentration
Icv: Intracerebroventricular
MAO: Monoamine oxygenase
MAOB: Monoamine oxidase B
MTDLs: Multi-target-directed ligands
MTT: 3(4,5Dimethylthiazol2yl)2,5 diphenyltetrazolium bromide
NFTs: Neurofibrillary tangles
NSAID: Non-steroidal anti-inflammatory drug
PAS: Peripheral anionic site
PLLA: Poly (l-lactic acid)
PLGA: Poly (lactide-co-glycolide)
RNS: Reactive nitrogen species
ROS: Reactive oxygen species
TAC: Tacrine
THA: Tetrahydroacridine
TrxR: Thioredoxin reductases
